# Modeling the Effect of Selection History on Pop-Out Visual Search

**DOI:** 10.1371/journal.pone.0089996

**Published:** 2014-03-03

**Authors:** Yuan-Chi Tseng, Joshua I. Glaser, Eamon Caddigan, Alejandro Lleras

**Affiliations:** 1 Beckman Institute, University of Illinois at Urbana-Champaign, Urbana, Illinois, United States of America; 2 Department of Mathematics, University of Illinois at Urbana-Champaign, Urbana, Illinois, United States of America; 3 Department of Psychology, University of Illinois at Urbana-Champaign, Urbana, Illinois, United States of America; 4 Department of Industrial Design, National Cheng Kung University, Tainan, Taiwan; 5 Interdepartmental Neuroscience Program, Northwestern University, Chicago, Illinois, United States of America; University of Bath, United Kingdom

## Abstract

While attentional effects in visual selection tasks have traditionally been assigned “top-down” or “bottom-up” origins, more recently it has been proposed that there are three major factors affecting visual selection: (1) physical salience, (2) current goals and (3) selection history. Here, we look further into *selection history* by investigating Priming of Pop-out (POP) and the Distractor Preview Effect (DPE), two inter-trial effects that demonstrate the influence of recent history on visual search performance. Using the Ratcliff diffusion model, we model observed saccadic selections from an oddball search experiment that included a mix of both POP and DPE conditions. We find that the Ratcliff diffusion model can effectively model the manner in which selection history affects current attentional control in visual inter-trial effects. The model evidence shows that bias regarding the current trial's most likely target color is the most critical parameter underlying the effect of selection history. Our results are consistent with the view that the 3-item color-oddball task used for POP and DPE experiments is best understood as an attentional decision making task.

## Introduction

Many studies have demonstrated that the attention system is extremely sensitive to recent experiences of attentional selection and deployment. For example, studies of the contextual cueing effect assert that the lingering effects of past experience implicitly shape the observer's selection bias and facilitate visual search [1], [2] and eye movements [3]. More recently, it has been shown that the reward associated with a recent context has an effect on the speed of implicit learning of that context [4]. Even the most efficient forms of salience-driven search, such as pop-out search (when a target feature differs uniquely from all distractors, and the target is always found equally quickly irrespective of the number of distractors in the display) are sensitive to recent experience. One demonstration is that responding to a red target among green distractors is faster on trial N, when there was the same target and distractor color assignment on trial N-1 (the Search Repeated condition), compared to when they switched assignments (i.e., the target was green among red distractors, the Search Switched condition [5]–[7]). This phenomenon is called Priming of Pop-out (POP), and the improvement in search performance is measured both in reaction times (RTs) becoming faster and accuracy increasing on Search Repeated trials compared to Search Switched trials [5], [6], [8]–[19]. The Distractor Preview Effect (DPE) [20]–[24], in contrast, describes how search performance deteriorates (i.e. search becomes slower and less accurate) when the current target features were associated with non-target status on the preceding trial. For example, responding to a red target among green distractors is slower on trial N, when on trial N-1 all objects were red (red was associated with non-target status) compared to when all objects were green.

This evidence suggests that the human brain has evolved an efficient visual system to find targets in the environment by shifting attention and eye gaze toward task relevant information around the observer. Traditional literature has shown two factors engaged in this control of attention: (1) the observer's goals, expectations, and biases (top-down factors) [25]–[27], and (2) the salience of objects in the world (a bottom-up factor) (e.g. [25], [28]–[33]). Awh, Belopolsky and Theeuwes [34] pointed out that the effect of selection history could conflict with the observer's goals [35]–[38]. They have thus suggested that *selection history* is an independent factor underlying the modulations observed in attentional selection, separate from the observer's explicit goal and the stimulus's physical salience [34].

Priming of Pop-out and the Distractor Preview Effect are both effects primarily caused by changes in selection history. The physical salience of the display does not change from trial to trial. Likewise, the observer's explicit goal always remains the same at the initiation of a trial (i.e. finding an oddball). Thus, here we look at a task with both POP and DPE conditions in order to better understand the effects of selection history through modeling. In this task, subjects need to make a saccade to an oddball color (when present). We can generally ask, given a simple two color display, how does selection history affect the decision of which color to select?

It is important to note that previous research has shown that the critical factor underlining these two effects is the stage during which target/distractor color assignments takes place, that is, the process through which a color (say, green) is tagged as being the color containing the target (in the current trial), while the other color (say, red) is tagged as the color of the distractors. Thus, even though in the surface the task may appear to be a “location” selection task (which of the items in the display should the saccade be directed to) it is in fact a “color” selection task (which of the two colors is the target color). Once the target color is found, saccading to the target is trivial. With respect to the DPE, we know location is unimportant from (at least) three previous studies. In Goolsby, Grabowecky and Suzuki [12], where the DPE was first documented, it was shown that changes to spatial features in the previous trial (the “preview trial”), like size of the items, number of items in the previous trial, and eccentricity of the items, had no effect on the magnitude of the DPE on the target-present trial. Later, Lleras, Levinthal and Kawahara [21] demonstrated that the DPE could be observed in a rapid serial visual presentation (RSVP) task, in which items are presented sequentially in a single (unchanging) location. On trial *N*-1, all items are of the same color, while on trial *N*, one of the items has a different color (is an oddball). As all items are presented at the same location, the DPE is likely not dependent on the spatial positioning of the to-be inspected items. Finally, and more crucially, Levinthal and Lleras [20] demonstrated that when RSVP trials (all search items in the same location, presented sequentially) are intermixed with spatial DPE trials (three items presented simultaneously at three different locations), an identical “spatial” DPE is found when the previous trial was spatial (three items/three locations) compared to when it was an RSVP sequence, showing that the spatial characteristic of the previous trial had no impact on the DPE. Moreover, they found an identical “single-location” DPE (in RSVP) when the previous trial was an RSVP sequence compared to when the previous trial was spatial, indicating that the DPE emerges from a difficulty in selecting a specific color as being the target color (and not with moving attention to a location), given that all items were presented at the same location. Finally, it should be noted that in 2010, Yashar and Lamy [17] found an identical result with POP. Thus, decision making regarding the color associated with target status (not the location containing the target) is at the crux of these two phenomena. It should also be noted that this is consistent with the top-down instructions to the participants, who must find the odd-colored item (not the odd-placed item), making color, not location, the defining attribute of the target in these tasks.

The decision in this sort of forced two-choice task is conventionally studied within the framework of a diffusion model. Ratcliff [39] developed a diffusion model (Ratcliff diffusion model; RDM) that turns the sampling of evidence into a noisy, ongoing evidence accumulation process. That is, evidence is sampled and accumulated at each point in time. After some number of samples (i.e., after some time), the accumulation of evidence can reach one of two thresholds: the signal threshold or the noise threshold. Once one of these criteria is reached, the decision is made to categorize the perceptual evidence as signal or noise. Importantly, the time course of the decision process can be predicted and modeled in different situations. The power of this approach is that one can make detailed predictions of human performance including predicting RT distributions for correct and error responses [39]–[42]. Therefore, this “diffusion” modeling approach has gained a lot of popularity in recent years.

An advantage of the RDM (and other accumulator models) is that it clearly separates different sources of variability in the model results. In the RDM, differing selection results can be due to changes in 3 central parameters: (1) the strength of incoming sensory information, (2) the boundary separation, representing the speed-accuracy tradeoff (SATO) strategy in the decision, and (3) the tendency bias, representing an initial decision tendency regarding the likely target color in the current trial. Finding the parameter changes responsible for POP and the DPE will shed light on how selection history affects attentional selection in the current trial.

We must clarify that we are agnostic as to whether these subjective beliefs (tendency bias) are explicit or implicit. In fact, previous research on the DPE has shown that cueing each trial ahead of time with information telling observers with 100% certainty whether or not the next trial will have a target does not affect the magnitude of the DPE [12]. Kristjansson, et al. [43] studied patients with unilateral spatial neglect and found POP can occur without awareness. Moreover, with healthy individuals, POP has been demonstrated to be contingent on the conscious perception of the target and implicit memory system [44]. Thus, it is quite likely that these beliefs may be implicit or explicit.

In this study, we aim to determine whether the Ratcliff diffusion model can successfully account for the changes in RTs and accuracy brought about by changes in selection history, specifically by looking at Priming of Pop-out [5]–[7] and the Distractor Preview Effect [22], [24], [45]. If this is the case, we expect to discover, from the model fitting, which factors (parameters in the model) are critical in the attention selection decision process underlying the effect of POP and the DPE.

## Methods

### Experimental Methods

All co-authors of this study affirm that the research was conducted in accordance with the principles expressed in the Declaration of Helsinki. Approval to conduct this research was granted by the University of Illinois at Urbana-Champaign, Office of the Vice Chancellor for Research, Institutional Review Board (IRB). Written Informed consent was obtained from all participants before the experiments were conducted.

In classic POP and DPE experiments, subjects were asked to find an oddball target and report a secondary feature of the oddball. For example, subjects needed to find the odd color diamond and then report the direction (left or right) of a missing corner in this diamond [5], [22], [24]. In the present study, we adapted these POP and DPE procedures to a saccade selection task in which participants were simply asked to make a decision on whether to saccade towards an oddball item (the color singleton) if one was present, or hold the eyes at fixation when there was no oddball item in the display [45]. After fixating the oddball, participants were asked to press a button to complete the trial.

Saccade selection tasks have been used previously in oddball tasks and many two alternative choice tasks [9], [10], [45]–[47]. It was used in the present study because it requires fewer processing stages than a manual response selection task. A saccade does not involve the motor process of a hand, but more importantly, it avoids a second decision about the cut-off side of a diamond. Thus, observing eye-behavior in a saccade generation task allows us to closely follow attentional decision making in the task [48]. We expected all subjects to show both Priming of Pop-out and Distractor Preview Effects in our saccade selection task when responding to the oddball [45], [46].

#### Participants

Five students (P1, P2, P3, P4 and P5) from the University of Illinois at Urbana-Champaign participated in this experiment in exchange for monetary compensation. Each subject participated in 5 sessions, and each session consisted of 5 blocks of 64 trials each. Therefore, there were 1600 trials for each participant in total. Each session lasted about 50 minutes. Three undergraduate students (P1, P2, and P5) had normal vision and two (P3 and P4) had corrected-to-normal vision. We opted for a psychophysical procedure (few subjects, many trials), so that the data could be modeled effectively.

#### Apparatus

Eye movements were monitored through an EyeLink 1000 system. A drift correction procedure was performed between trials to ensure spatial accuracy of eye movement data. Trials in which the fixation landed within 2 degrees of the target (i.e. oddball) were classified as correct, and trials in which the fixation landed within 2 degrees of the distractor were classified as incorrect. Trials were excluded from analysis in the following conditions: blinks, failure to fixate on the center dot at the beginning of the trial, or failure to saccade within 2 degrees of any object on the display during target-present trials. Stimuli were presented on a 21-inch CRT monitor at a resolution of 1024×768, at 85 Hz. We recorded saccade latency, reaction time (time to press the button to advance to next trial), and whether the initial saccade was correct or not (i.e., whether it landed on the oddball target or elsewhere).

#### Stimuli

**Figure 1:**
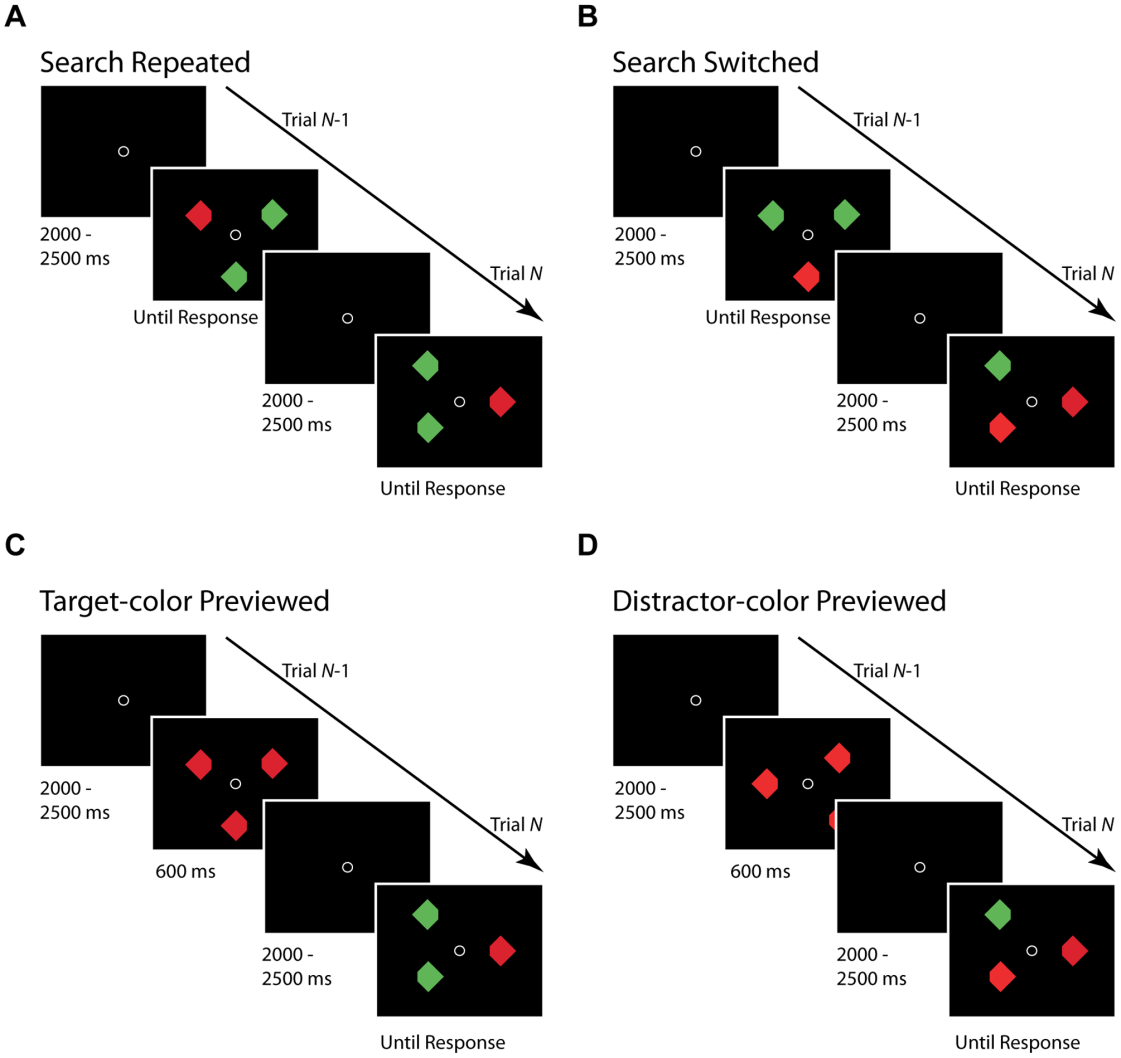
Samples of Four Search Conditions (Target Is Red and Two Distractors Are Green in Current Trial). (a) Search Repeated (target color and distractor color are repeated across consecutive trials); (b) Search Switched (target color and distractor color are switched across consecutive trials); (c) Target-color Previewed (the current target color was the color of objects in the preceding non-target trial); and (d) Distractor-color Previewed (the current distractor color was the color of objects in the preceding non-target trial). Search Repeated and Search Switched conditions comprise the POP search task, and Target-color Previewed and Distractor-color Previewed conditions comprise the DPE search task.

Figure 1 depicts the stimuli and trial sequence in our four search conditions. The stimuli and configurations used in the present experiment were based on those used by Goolsby and colleagues [12] and later by Caddigan and Lleras [45]. Three diamonds were placed over an iso-accuity ellipse centered at fixation, and with an equal central angle between each. There were 12 locations on the ellipse. Therefore, there were four different 3-item display configurations. The target itself was randomly located at each of the three locations in any given configuration. Diamonds were either green or red, and on each trial, participants were instructed to make a saccade towards the oddball if one was present. Once their eye reached the target diamond, they pressed a button to finish the trial. The oddball color was equally likely to be either red or green, so participants did not know what color the target would be at the onset of the trial.

#### Trial Sequence and Procedure

A pseudorandom sequence of trials was generated as by previous studies [24], [45]. To generate each set of 32 trials, eight trial pairs (16 trials) appeared in random order, representing the Search Repeated, Search Switched, Distractor-color Previewed, and Target-color Previewed conditions, with each color (green or red) used as a distractor. Because all trials in the pairs of Search Repeated and Search Switched conditions are target-present, while half of trials in the pairs of Distractor-color Previewed and Target-color Previewed conditions are target-present, there were 12 target-present trials (8 from the POP condition and 4 from the DPE condition) and 4 target-absent trials. An additional 16 trials were then randomly inserted in between these trial pairs; half of these were target-absent trials, and each color (green or red) appeared as a distractor four times for each condition. This process resulted in variable numbers of target-absent trials and target-present trials. The number of trials in each of the four inter-trial search conditions was also variable. When a target-absent trial was inserted randomly before a target-present trial, this could create a Distractor-color Previewed or Target-color Previewed trial (eliciting a Distractor Preview Effect); when a target-present trial was followed by another target-present trial, this would add an additional Search Previewed or Search Switched trial (subject to Priming of Pop-out).

Each trial started with the presentation of a central fixation point. After a variable interval of 2000–2500 ms, the search display appeared. In the target-absent condition, the search display was presented for 600 ms, and subjects were expected to maintain fixation throughout that interval. On target-present trials, the search display remained visible until participants pressed the button to indicate the end of the trial. We analyzed the data on a given trial with respect to its relation to the preceding trial. In the “Search Repeated” condition (SRe), the color of target and distractors repeated across consecutive trials, Figure 1a; mean  = 32 trials/session. In the “Search Switched” condition, the color assignment between target and distractors on the current trial was opposite to that of the previous trial (so, if on the previous trial, a red target amongst green distractors was seen, there would be a green target amongst red distractors on the current trial), Figure 1b; mean  = 36 trials/session. In the “Target-color Previewed condition” (TP), the color of distractors on the preceding target-absent trial became the color of the target on the current target-present trial, Figure 1c; mean  = 26 trials/session. Finally, in the “Distractor-color Previewed” condition (DP), the color of distractors on the preceding target-absent trial was the same as the color of distractors on the subsequent target-present trial, Figure 1d; mean  = 24 trials/session.

### Modeling Methods

The goal of our participants was to saccade to the color oddball target as soon as possible (if present). Under our experiment structure (the target is defined as either red or green in the whole experiment), our participants first had to decide which of the two colors was the target color. To model this task, we used the Ratcliff Diffusion Model (RDM) [39], which models the decision making process between two options. This model can shed light on which parameters are critical for the DPE and POP, and more generally for the effect of selection history.

**Figure 2:**
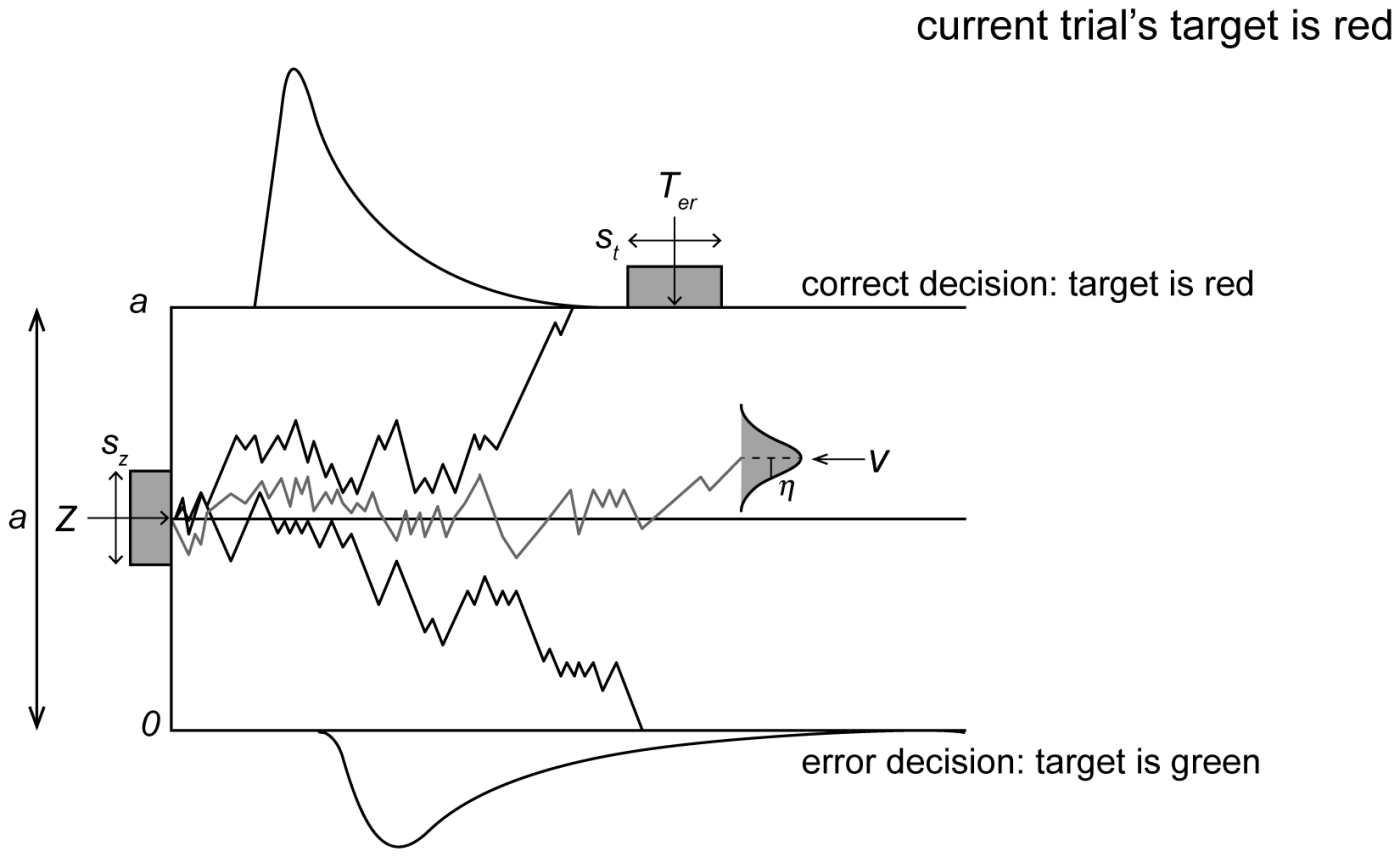
An Illustration of the Ratcliff Diffusion Model. Evidence starts accumulating at starting point *z* (inter-trial variation *S_z_*). It accumulates with a drift rate mean of *v* and standard deviation of *s* (a scaling parameter not shown). The mean drift rate has an inter-trial variation of *η*. The evidence accumulates towards one of two decision thresholds, which are separated by the boundary separation *a*. An additional parameter is the non-decision time, *T_er_* (inter-trial variation *S_t_*).

In the RDM framework (Figure 2), the initial value of belief regarding which color the target may be starts at a baseline level, *z*, and accumulates towards one of two thresholds. These thresholds represent the level of belief required to make a decision that a color (green or red in our experiment) is the target in the trial. One threshold is at a level of 0, and the other at *a*. Evidence accumulates with a drift rate mean of *v* and standard deviation of *s* (this is a scaling parameter that we set at 1). When the evidence reaches a threshold, a decision regarding target color is made in favor of the corresponding color threshold. As the entire reaction time does not consist solely of the decision-making time, there is also a parameter *T_er_*, which represents time for processes other than decision-making. The RDM parameters combine to produce an estimate of sensory evidence accumulation in our tasks.

In our task, we always set the current trial's target's color at threshold *a (target-color threshold)*, and the distractor's color at threshold 0 *(distractor-color threshold)*. Thus, evidence that accumulates to *a* leads to a saccade towards the target (i.e. correct saccade). Evidence that accumulates to 0 leads to a saccade towards one of the distractors (i.e. incorrect saccade). The target color may be red or green, and we combine these scenarios for modeling. As an example, if a trial's target is red in the “Search Repeated” (SRe) condition of POP, the target color (red) competes with the distractor color (green) to be decided as the target (see Figure 1). When the accumulation of evidence reaches a confident value of implicit belief (i.e. *a*, the target-color threshold, red in this example) that red is the target, a decision is made, followed by a correct saccade. However, when the accumulation of evidence reaches a confident value of implicit belief (i.e. 0, the distractor-color threshold, green in this example) that green is the saccade target, a decision is made, followed by an error saccade. The accumulator value at any time can be viewed as a comparison of the accumulated evidence towards the target color versus the accumulated evidence of the distractor color (in the above example, the accumulator will be closer to *a*, at any given time if there is more accumulated evidence that red is the target color).

The starting point *z* represents an estimate of biases towards a certain color being the target in our tasks. When *z* = *a/*2, there is no bias, as the start point is halfway between the thresholds. Values of *z>a/*2 represent a bias towards target color, and *z*<*a*/2 represents a bias towards the distractor color. Thus, *z*/*a*, which we will refer to as *B* from now on, gives a true estimate of the bias in a trial. The drift rate, *v*, represents the comparative quality of sensory information from the stimuli. A positive *v* signifies that the target has a stronger signal in the display than the distractors (as it should be when it is a pop-out target). A *v* with a larger magnitude signifies a greater signal-to-noise ratio of the stimulus. The boundary separation, *a*, relates to the speed and accuracy tradeoff strategy (SATO strategy) of the decision to saccade. For example, when the boundary separation is small, although the decision process can hit the target boundary quicker, there is also a greater chance that the process may hit the wrong threshold. Lastly, as mentioned previously, *T_er_* represents the non-decision time. This can include processes such as stimulus encoding and motor response delay. Together, understanding the parameters *B, a*, *v, and T_er_* can aid in understanding the underlying mechanisms of choice tasks.

It is important to note that several other parameters are included in the RDM in order to better fit empirical data. *S_z_*, *S_t_*, and *η* are parameters that relate to inter-trial variability. *S_z_* is the range of *z* across trials, *S_t_* is the range of *T_er_* across trials, and *η* is the standard deviation of *v* across trials.

#### Parameter Constraints

For each subject, *S_z_*, *S_t_*, and *η* were always constrained to be the same across all experimental conditions, in order to focus solely on the change in *T_er_, z, a*, and *v* across conditions. Additional parameter constraints were necessary to reflect the possible inter-trial modifications. For instance, if on trial *N-1*, the display was all green (green was a distractor color), then prior to trial *N*, there may be a bias against the color green. This would be reflected as a bias away from the target if the current display (trial *N*) contains a green target (Target-previewed Condition, TP), or a bias towards the target if the current display (trial *N*) contains a red target (Distractor-color Previewed condition, DP). Thus, the bias, *B*, for the TP condition is 0.5-▵*B*_DPE_, while *B = *0.5+▵*B*_DPE_ for the DP condition. Recall that *B* = *z/a* = *0.*5 signifies an unbiased starting point, one that is equidistant from the target and distractor thresholds. Similarly, if on trial *N-1*, the target was red, then prior to trial *N*, there may be a bias against the color green, regardless of whether the next trial has a green target or distractors. Thus, for the search repeated condition, *B = *0.5+▵*B*_POP_ while for the search switched condition, *B = *0.5-▵*B*_POP_. A similar logic is used to constrain the drift rate, *v*, across conditions. A subject has a baseline drift rate, *v_0_,* that can be modified by the inter-trial effects through the sensory processing. For the DP, TP, SRe, and SSw conditions, the drift rates are *v_0_*+▵*v*_DPE_, *v_0_*-▵*v*_DPE_, *v_0_*+▵*v*_POP_, and *v_0_*-▵*v*_POP_, respectively. Note that all ▵*B*'s and ▵*v*'s can be either positive or negative. Also, based on the assumption that parameter modifications from these inter-trial effects happen between trials as opposed to during the current trial, the threshold separation, *a*, should be identical between a DP and TP condition (see Figure 1a, b) or between SRe and SSw condition (see Figure 1c, d) since the preceding trial was identical. The same is true for the non-decision time, *T_er_*. This gives us the parameters *a_DPE_, a_POP_, T_DPE_*, and *T_POP_*. In total, we are fitting a total of 12 model parameters: ▵*B*_DPE_, ▵*B*_POP_, *v_0_*, ▵*v*_DPE_, ▵*v*_POP_, *a_DPE_, a_POP_, T_DPE_, T_POP_, S_z_*, *S_t_*, and *η*.

#### Fitting the RDM

The model was fit to the observed saccade latency and accuracy data in each condition for each individual participant. Our model was fit similar to Ratcliff and Tuerlinckx's Chi-Square Fitting Method [49]. For each experimental condition, for correct saccades, our saccade latency data was split into six bins and then the model's saccade latencies were compared to subjects' data in each bin to compute a chi-square value. The points at which bins are split are referred to as quantiles, and were set at 10%, 30%, 50%, 70%, and 90% of the cumulative reaction time distribution. Due to our very limited number of error trials (see Table 1), only one chi-square value, as opposed to six, was computed for error trials, as done in [50]. Finally, we summed all these chi-squared values across conditions. We had 28 chi-square values (four conditions times seven values per condition) that were summed to get the total chi-square value. We found the parameters that minimized this chi-square value. Note that this method differed from Ratcliff and Tuerlinckx's method [49] only in the number of error bins, and multiple studies have expressed issues with the original method for high-accuracy experiments. It should be noted that with using only 1 bin, the error chi-squared value had to be excluded for subjects P1, P2, and P4 in the DP condition (there was 100% accuracy). Additionally, for each subject, trials that had reaction times more than 5 standard deviations away from the mean were considered outliers and excluded from the fitting. We fit our model using the Diffusion Model Analysis Toolbox [51]. Modifications to the toolbox were made in order to have one error bin and in order to allow the constraint format of the bias parameter.

**Table 1:** The Mean Saccade Latency and Accuracy in Four Search Conditions in Each Subject.

		SRe	SSw	p		TP	DP	p
P1	latency (ms)	279.2	301.5	<0.001		305.8	288.2	<0.001
	accuracy (%)	98.1	94.0	0.024		92.0	100.0	<0.001
P2	latency (ms)	304.0	320.3	0.049		332.3	295.1	<0.001
	accuracy (%)	98.5	95.1	0.106		94.6	100.0	0.016
P3	latency (ms)	285.9	293.6	0.233		332.6	307.8	0.010
	accuracy (%)	94.4	79.2	<0.001		88.1	97.2	0.004
P4	latency (ms)	374.8	389.9	0.016		408.5	390.7	0.165
	accuracy (%)	98.7	98.9	0.869		97.3	100.0	0.092
P5	latency (ms)	315.3	331.4	<0.001		329.7	315.9	0.076
	accuracy (%)	98.3	96.9	0.276		97.5	99.4	0.170

P-values were calculated using a two-tailed t-test for latency and a chi-square test for accuracy.

#### Analysis of Parameter Fits

To analytically determine whether the model fit to the data was acceptable, we determined a critical chi-squared value that our best-fit chi-squared values could be compared with, as done in [52], [53]. That is, we determined the chi-squared value that would cause our model to be rejected at a significance level of 0.05. Thus, if the chi-squared value for a model fit was less than the computed critical value, then that model cannot be rejected. This chi-squared comparison was done for each subject. The degrees of freedom of the chi-squared value is J*(K-1)-M, where *J* is the number of conditions (4 in our case), *K* is the number of chi-squared values computed per condition (7 in our case), and *M* is the number of model parameters. Note that for subjects P1, P2, and P4, there is one fewer degree of freedom because no chi-squared value was computed for errors in the DP condition.

We also show the quality of fits using quantile probability plots [49]. These plots simultaneously show the average reaction time and accuracy at each quantile for every experimental condition for both the model and the experimental data, and thus are an excellent way of visualizing the fit of the model. Note that because we only use quantiles for correct trials, only correct trials are shown in our plots. We separately show the number of errors predicted by the model versus the experimental data for each experimental condition for each subject.

#### Optimal Sub-Model of the RDM

In order to provide a first analysis of the RDM parameters critical to the phenomena of the DPE and POP, we compare several “sub-models” in which one or two additional parameters were constrained between conditions, in order to find the best sub-model. That is, will constraining certain parameters cause the quality of fits to drastically decrease, and will constraining other parameters have minimal effect on the model fits? This model comparison allowed us to identify the critical parameters for modeling these effects (i.e., the parameters without which fitting becomes poor). We compared the 12-parameter model, which we will refer to as the “full” model, to 8 other models where parameters were constrained across all experimental conditions: 1) no bias in all trials- ▵*B*_DPE_ = 0 and ▵*B*_POP_ = 0, so for all conditions *B* = 0.5; 2) no bias in DPE trials- ▵*B*_DPE_ = 0; 3) no bias in POP trials- ▵*B*_POP_ = 0; 4) drift rate doesn't change in all trials- ▵*v*_DPE_ = 0 and ▵*v*_POP_ = 0, so for all conditions *v* = *v*_0_; 5) drift rate doesn't change in DPE trials- ▵*v*_DPE_ = 0; 6) drift rate doesn't change in POP trials- ▵*v*_POP_ = 0; 7) thresholds are equal in POP and DPE trials- *a_DPE_* = *a_POP_*; 8) non-decision times are equal in POP and DPE trials- *T_DPE_* = *T_POP_*. These models are compared using the Akaike information criterion (AIC) and Bayesian information criterion (BIC). These metrics compute the likelihood of the model fit to the experimental data and penalize the number of parameters used in the model (the model's complexity). Quantitatively, AIC = −2ln(*L*)+2*M*, where *L* is the likelihood of the model, and *M* is the number of parameters in the model [54]. BIC = −2ln(*L*)+M*ln(*n*), where *n* is the number of observed data points [55]. Both metrics are included because AIC has a bias towards larger models [56], while BIC has a bias towards smaller models [57]. For every subject, the AIC and BIC of the sub-models are compared to the full model to determine the importance of a parameter for a good model fit. For an across subjects comparison, we add the AIC/BICs, which is equivalent to finding the likelihood of the data across all subjects (assuming subjects are independent) with the aforementioned model complexity penalty.

#### Parameters of the RDM

Finally, we looked into the best-fitting parameters of the full model. Are there parameter trends that are consistent across subjects? We tested whether bias and/or sensory parameter (i.e. drift rate) changes responsible for DPE and POP (▵*B*_DPE_>0, ▵*B*_POP_>0, ▵*v*_DPE_>0, ▵*v*_POP_>0) using a one-tailed t-test. We tested whether thresholds and non-decision times are different between DPE and POP trials (*a_DPE_*≠*a_POP_*, and *T_DPE_*≠*T_POP_*) using a two-tailed paired t-test.

## Results

### Experimental Results

We scored a trial as correct if the initial saccade landed within 2 degrees of the oddball target and incorrect if it landed within 2 degrees of a distractor. Saccade latency was the time elapsed between the onset of the search display and the first saccade with a magnitude greater than 2 degrees.

#### Priming of Pop-Out

Table 1 shows search performance across all four conditions for each individual. All five participants had a shorter saccade latency in the Search Repeated (SRe) condition than in the Search Switched (SSw) condition (all *p*'s<0.05 except P3; two-tailed t-test). These results confirm that generally participants showed Priming of Pop-out. Along with faster RTs, participants had higher accuracy rates in the Search Repeated condition than in the Search Switched condition, with the exception that P4, whose accuracy was nearly perfect in both conditions (98.7% and 98.9% in SRe and SSw condition, respectively). This difference was significant for P1 and P3 (chi-square test). In sum, generally our participants made a saccade to foveate the color oddball faster and more accurately when the target and distractor colors repeated across trials than when they switched.

#### Distractor Preview Effect

To examine the DPE, we compared Distractor-color Previewed (DP) trials and Target-color Previewed (TP) trials. Saccade latencies were longer for all five participants in the Target-color Previewed condition than in the Distractor-color Previewed condition, as expected (Table 1). This difference was significant in P1, P2 and P3, and marginally significant in P5 (two-tailed t-test). Along with longer saccade latencies, accuracy was also lower on TP than on DP trials for all subjects. This difference was significant for P1, P2, and P3 (chi-square test). Overall, this result shows that, when asked to saccade to a visual oddball, participants were slower to do so (and less accurate) when the color of the current target was the color of distractors on the previous target-absent trial. Observers had a measurable difficulty in foveating the pop-out under these conditions (replicating [45]). It is worth noting that the Target-color Previewed condition was the condition associated with the worst level of performance across all subjects and all four conditions: it consistently yielded the longest observed saccade latencies. Finally, we also measured the frequency of false alarms in the experiment, defined as saccades that were executed on target-absent trials, that is, the proportion of target-absent trials in which participants failed to maintain fixation on the center of the screen. For subjects P1, P2, P3, P4 and P5, saccades were erroneously made on these trials 0.94%, 4.84%, 10.59%, 0.98% and 0.62% of the time, respectively.

### Modeling Results

In the experiment, changes in saccadic latencies demonstrated that every individual was affected by POP and the DPE. The saccade selection task in the present study allowed us to then model how these different types of recent experiences (from the preceding trial) modulate the decision process of deploying attention to a pop-out item.

#### Analysis of Parameter Fits

To quantitatively determine whether our parameter fits were acceptable, we compared the chi-squared values of each subject's model fit (from the full model) to a critical chi-squared value. If the value from the model fit is less than the critical value, then the fit is deemed acceptable. For subjects P3 and P5, the critical chi-squared value was 21.026 (12 df). For subjects P1, P2, and P4, the critical chi-squared value was 19.675 (11 df). Table 2 shows that 4 out of 5 subjects have model fit chi-squared values below the critical value, and P3 has a value (23.202) slightly above the critical value of P3, 21.026. This demonstrates that overall, the RDM provides acceptable fits to POP and DPE data. P3 had the worst accuracy compared to other subjects, particularly in SSw, which may be the reason why that the model could not fit P3 as well as the other participants.

**Table 2:** Best-Fit Parameters of Full Model.

	P1	P2	P3	P4	P5	(Mean, SEM)
χ^2^	8.617	14.928	23.202	3.693	18.871	(13.862, 3.496)
▵*v*_DPE_	−0.0839	−0.0102	0.0187	0.0798	0.0201	(0.0049, 0.0266)
▵*v*_POP_	−0.0168	0.0017	0.0582	−0.1287	−0.0187	(−0.0209, 0.0303)
▵*B*_DPE_	0.1852	0.1560	0.1038	−0.0517	0.0621	(0.0911, 0.0415)
▵*B*_POP_	0.1437	0.0960	0.0889	0.1379	0.0666	(0.1066, 0.0148)
*a_DPE_*	0.0567	0.0749	0.1229	0.0749	0.0916	(0.0842, 0.0111)
*a_POP_*	0.0640	0.0759	0.0832	0.0941	0.0933	(0.0821, 0.0056)
*T*_DPE_	0.2579	0.2497	0.2313	0.3275	0.2530	(0.2639, 0.0165)
*T*_POP_	0.2466	0.2419	0.2415	0.3052	0.2537	(0.2578, 0.0121)
*v*_0_	0.6785	0.5084	0.7754	0.5854	0.6466	(0.6389, 0.0448)
*S_z_*	0.0000	0.0000	0.0674	0.0000	0.0623	(0.0259, 0.0159)
*S_t_*	0.0467	0.0726	0.0490	0.0903	0.0157	(0.0549, 0.0127)
*η*	0.2083	0.0000	0.5000	0.1439	0.0000	(0.1704, 0.0919)

**Figure 3:**
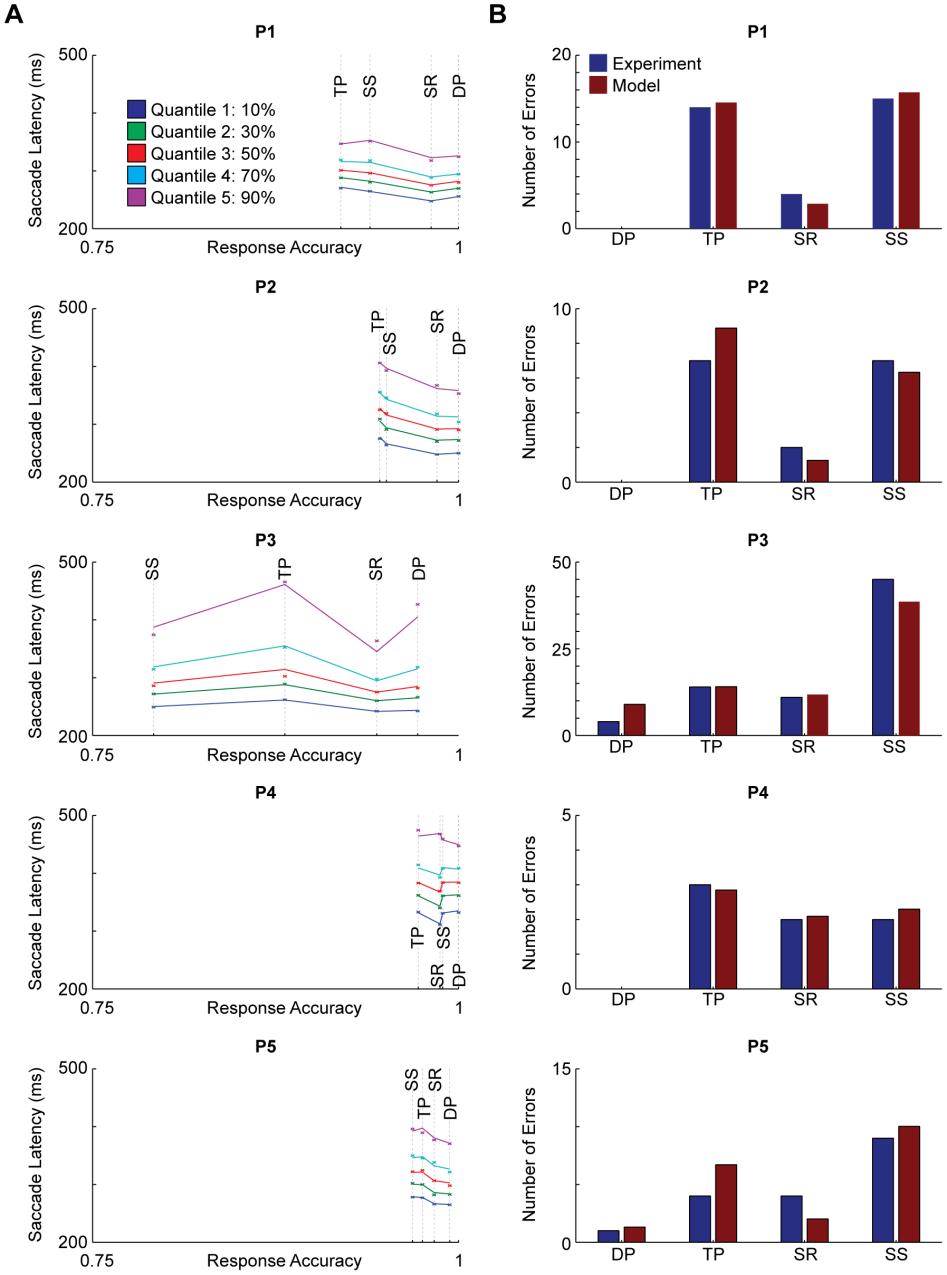
Model Goodness of Fit. (a) Quantile probability plots for the correct trials. These compare reaction time distributions from the model (lines) with empirical reaction times (x's) for every experimental condition. (b) Number of error trials generated by the model (red) is compared to the number of empirical error trials (blue) for every experimental condition.

To look more in depth at the quality of fits, we provide quantile probability plots (QPPs; Figure 3a) for correct trials and a comparison of the number of error trials (Figure 3b). Overall, we find that there are qualitatively very good fits, with the exception of the model not fitting the data very well for error trials in subject P5 and the top quantile (90%) of RTs of correct trials in subject P3.

#### Optimal Sub-Model of the RDM

**Figure 4:**
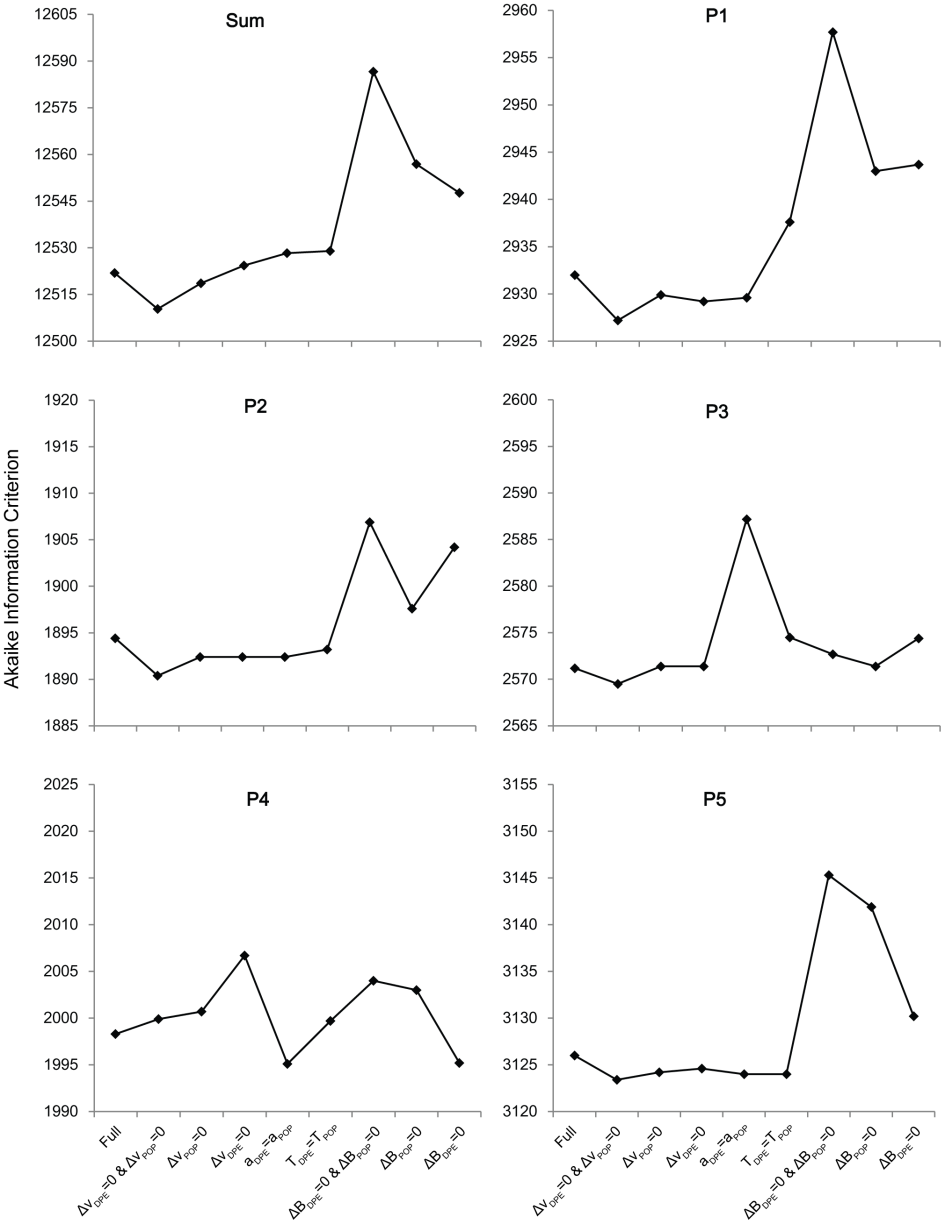
AIC Model Comparison. Akaike information criterion (AIC) values are compared between several sub-models of the RDM. Each sub-model has one or two constrained parameters compared with the full model. This comparison is done for every subject, and the sum of AIC values is used for an across-subjects comparison. Note that AIC biases model selection towards models with more parameters.

**Figure 5:**
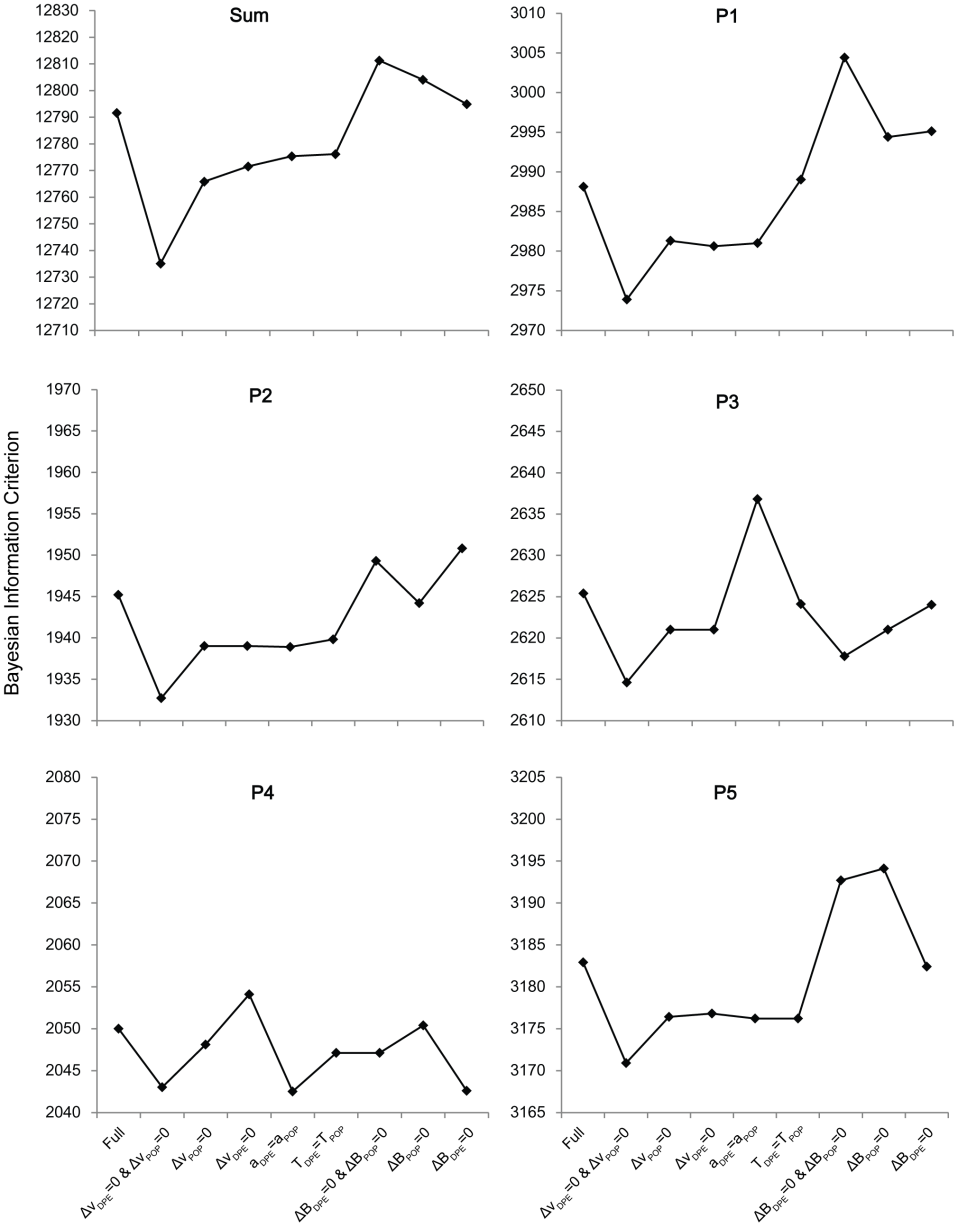
BIC Model Comparison. Bayesian information criterion (BIC) values are compared between several sub-models of the RDM. Each sub-model has one or two constrained parameters compared with the full model. This comparison is done for every subject, and the sum of BIC values is used for an across-subjects comparison. Note that BIC biases model selection towards models with fewer parameters.

In order to determine the parameters of the RDM critical for modeling POP and the DPE, we compared models that constrain these parameters across conditions using the Akaike information criterion (AIC; Figure 4) and Bayesian information criterion (BIC; Figure 5). Lower AIC/BIC values signify a better model fit. A model comparison demonstrates that the sub-model with ▵*v*_DPE_ = 0 and ▵*v*_POP_ = 0 outperforms the full model. The AIC values for the v-constrained model (▵*v*_DPE_ = 0 and ▵*v*_POP_ = 0) are less than the full model for 4 of 5 subjects (not P4) and have the lowest summed AIC value. As AIC comparisons have a bias towards larger models, the fact that the smaller (v-constrained) model has lower AIC values is robust. Thus, the change in drift rate is likely to be an unimportant parameter when modeling the inter-trial effects of the DPE and POP.

The model with ▵*B*_DPE_ = 0 and ▵*B*_POP_ = 0 is consistently worse than the full model. It has AIC values higher than the full model in all 5 subjects and has the largest summed AIC value. As AIC is biased towards larger models, we also compare the BIC values, which have a bias towards smaller models. Still, even with this bias towards the B-constrained (Bias is constrained) model, the summed BIC is larger than the full model, and is larger in the majority of subjects. Together, the AIC and BIC results suggest that bias is a critical model parameter when modeling POP and DPE.

The model with *a_DPE_* = *a_POP_* has a lower AIC value than the full model in 4 of 5 subjects, but has a larger summed value than the full model. However, this model has a smaller summed BIC value than the full model, and still, 4 of 5 subjects have a lower BIC value than the full model. The model with *T_DPE_* = *T_POP_* has a lower AIC value than the full model in 2 of 5 subjects (P2 and P5) and has a larger summed value than the full model. However, this model has a lower BIC value than the full model in 4 of 5 subjects and has a smaller summed value than the full model. Thus, it is not yet clear whether the a-constrained and T-constrained models are better than the full model, and whether these parameters vary between the DPE and POP conditions.

#### Parameters of the RDM

To further investigate the parameters of the RDM critical to effectively modeling the DPE and POP, we looked for parameter trends across subjects in our full model (Table 2). We first tested whether a bias is important in accounting for the DPE and POP, meaning testing whether ▵*B*_DPE_>0 and ▵*B*_POP_>0. ▵*B*_POP_>0 in all 5 subjects and this ▵*B*_POP_>0 is significant (p = 0.001; one-tailed t-test). ▵*B*_DPE_>0 in 4 of 5 subjects and is significant (p = 0.047; one-tailed t-test). These results support the model comparison results suggesting that bias, ▵*B*, is a necessary parameter in models of both POP and the DPE.

A change in drift rate could also account for the inter-trial effects, so we tested whether ▵*v*_DPE_>0 and ▵*v*_POP_>0. However, ▵*v*_DPE_>0 and ▵*v*_POP_>0 are true in only 3 and 2 subjects, respectively. Both test results are insignificant. These results again support the model comparison results; a change in drift rate is unlikely the cause of the DPE and POP.

We also tested whether there is a difference in both boundary separations and non-decision times between the DPE and POP conditions, that is, whether *a_DPE_* = *a_POP_* and *T_DPE_* = *T_POP_*. The best fit results show that *a_POP_*>*a_DPE_* in 4 of 5 subjects, but the test for whether *a_DPE_*≠*a_POP_* is insignificant (p = 0.97; two-tailed t-test). *T_DPE_*>*T_POP_* in 3 of 5 subjects, and the test for whether *T_DPE_*≠*T_POP_* is insignificant (p = 0.31; two-tailed t-test). These results show that the boundary separation and non-decision times are not consistently different between DPE and POP conditions, although there are large individual variations.

Note that all these parameter trends can also be found in a model where *S_z_*, *S_t_*, and *η* are allowed to be different in DPE and POP trials (Table S1). Additionally, none of these three additional parameters are consistently different between DPE and POP trials. Also, in the optimal submodel, where ▵*v*_DPE_ = 0 and ▵*v*_POP_ = 0, parameter trends (with the exception of the ▵*v*'s) are consistent with the full model (Table S2).

## Discussion

This study aimed to clarify the mechanisms underlying the attentional modulations caused by selection history, in particular by examining Priming of Pop-out (POP) and the Distractor Preview Effect (DPE). To do this, we fit the Ratcliff diffusion model (RDM) to eye movement data from an experiment that included a mix of POP and DPE search conditions where the oddball was defined by the item with a unique color in the search array. The experiment showed that a saccade to the target in trial *N* is faster when the current target color was also the target color in trial *N-1*, and it was slower when the current target color was the color of all three objects in trial *N-1* (i.e., there was no oddball and no saccade triggered). In other words, we replicated Priming of Pop-out [5]–[7] and the Distractor Preview Effect [20]–[24], [45] in an experiment that allowed us to model both of these effects in each participant. We first showed that the RDM can accurately model these inter-trial effects. The results of this modeling suggest that a bias (i.e. ▵*B* in the model) towards the current target's color (or away from the distractor's color) is a necessary parameter for both POP and the DPE to occur, while the change in the strength of the representation of the stimuli feature (▵*v*), boundary separation (*a*) and non-decision time (*T_er_*) are less likely the factors underlying of the DPE and POP. Finally, the success of the modeling implies that, indeed, these two tasks can be understood as attentional decision making tasks, in which the attention system must quickly decide the target/distractor status assignments to two colors presented in the display.

### Behavioral Correlates to RDM Parameters

In order to better understand the meaning of our results, it is useful to look at how the RDM parameters have been modulated in past experiments. When the relative proportions of stimuli vary between conditions, the bias parameter has changed most [58]. A previous disproportionate occurrence of conditions is an example of an effect of selection history, thus supporting our finding that selection history is primarily represented in the RDM through the bias parameter. Their study also found that minor modulations in drift rate also accompanied a change in stimuli proportions, while our study did not find any robust changes in drift rate during the DPE and POP. The drift rate parameter has been consistently shown to change due to changes in stimulus quality and discrimination ability (the relative bottom up salience of stimuli) [58], [59]. As the bottom-up salience of targets in all trials was constant across experiments, it is not surprising that we did not see a consistent effect on drift rate. The boundary separation alters due to emphasis on speed or accuracy (the speed accuracy tradeoff, SATO), and thus depends on task difficulty [58]–[60]. In our experiment, subjects' speed-accuracy goals remained consistent between conditions, and the fact that we found no robust difference in boundary separation suggests that DPE conditions are not intrinsically easier or harder than POP conditions. Lastly, the bias parameter has also shown to be modulated by differing reward rates for different conditions [59], which was not tested in our experiment. In sum, our finding that POP and the DPE, which are caused by differences in selection history, are dependent on changes in the bias parameter, is mostly consistent with previous experimental and modeling results.

### Neural Correlates for Priming of Pop-out and the Distractor Preview Effect

Single neuron recordings have been done in primates doing oddball search tasks in which Priming of Pop-out occurs [46], [61]. These studies showed that having the same target in the previous trial as the current trial (the search repeat condition) led to an increased firing rate in many frontal eye field (FEF) neurons compared to when the current trial's target was a distractor in the previous trial.

There have not previously been neural recordings of the Distractor Preview Effect. However, it is possible to make predictions given our modeling findings, as diffusion models and other related accumulator models have been shown to mimic neural activity [62]–[66], including the neural correlates of oddball visual search [67]. We found here that POP and the DPE can be explained in the RDM by a change in the same parameter, suggesting that the neural causes of the two effects may be similar as well.

POP and the DPE have both been investigated in humans using fMRI. In Kristjansson et al. [68], the authors examined the same color Pop-out task as we used here (although they did not include DPE trials). They found that the FEF and intraparietal sulcus (IPS) responded more when the target had changed color from one trial to the next, implying that the dorsal attention network was responsible for maintaining the change in belief about the likely target identity across trials. Complementing those results, recently Scalf, Ahn, Beck and Lleras [69] studied only DPE trials under fMRI, using categorical oddball displays (find house amongst faces or vice-versa). They found that the DPE was significantly associated with changes in the ventral attention system (bilateral IFG, right angular gyrus and right supramarginal gyrus, as well as the right middle frontal gyrus). This is interesting because contrasting the two sets of fMRI data one can conclude that POP and DPE “live” in different attention networks, even though our data suggests that both of these selection history effects are represented analogously, via changes in bias about the target color.

### Bias Modulation Is the Major Factor Underlying POP and the DPE

The modulation of the bias parameter could signify multiple internal effects. One possibility is that it could represent perceptual saliency (or perceptual gain). We believe this is unlikely, as perceptual saliency would most likely be represented in the same manner as physical saliency, i.e. through the drift rate parameter [58], [59]. Another possibility is that the bias parameter could represent a bias of belief. This is likely, as increasing the probability of a target, which has been shown to modulate the bias parameter [58], would change someone's internal belief about the likelihood of the target.

This parameter interpretation suggests that search facilitation in the SRe condition of POP is because of a positive bias effect: seeing the target color on a trial increases the bias of belief that the target will again be that specific color on the subsequent trial. This result is in line with accounts proposing no contributions of changes in perceptual salience as an underlying mechanism behind POP (e.g. [70]–[73]).

Similar to POP, our modeling results show a reliable modulation of bias in DPE.

Generally, the modeling results regarding the bias parameter in the DPE are well in agreement with a number of studies by Lleras and colleagues [20], [22], [45] showing that that DPE is a top-down attentional bias. This bias prevents attention from shifting towards features associated with target-absent status. Lleras, Kawahara and Levinthal [21] have suggested that the DPE can be very taken as an example of an *attentional repulsion effect* centered on the target itself (and its history): it is not so much the case that distractors attract more attention onto themselves on Target-Previewed trials (compared to DP), but rather that it is much harder to move attention towards the target on those trials because of the history associated with the target's color (i.e., its recent assignment of non-target status, see also Caddigan and Lleras [45]). Lleras et al. [21] studied a temporal search task version of the DPE studied here: participants were asked to find the color (or category) oddball on a rapid serial visual presentation (RSVP) task. Performance on the trial subsequent to a target-absent RSVP stream was near floor when participants had to select an item of the color of the items on the preceding target-absent RSVP stream (i.e., a Target-color Previewed condition, in RSVP presentation), whereas little difference was found between Distractor-color and Neither-color Previewed conditions. This pattern observed in the temporal version of the DPE is well in line with a “target-repulsion” account of the DPE. Interestingly, as noted in the *Introduction*, the repulsion bias “transfers” across search tasks: that is, when RSVP search trials are intermixed with spatial search trials (find the oddball in a static display), a DPE is observed irrespective of the type of target-absent trial type (RSVP or spatial), and irrespective of the target-present trial type [20]. This seems to also be the case in POP [17].

### The 3-Item Pop-Out Task Is an Attentional Decision Making Task

When viewed from this modeling framework, we propose that the task we have been studying in the POP literature may actually not be a strict “pop-out” task. By that, we mean that the three-item display used consistently in this literature may not carry sufficient information to truly induce a feature-based pop-out effect (i.e., an entirely bottom-up driven reaction to the display). In larger display sizes, the color-contrast signal between the target and the surrounding distractors may be sufficiently large to induce a bottom-up contrast that drives the eye to the pop-out (a true feature-based pop-out), but that may not be so for three-items displays. In fact, displays with larger set sizes do produce faster RTs than those with smaller set sizes [5], [74], and in fact, POP magnitude is smaller with larger set sizes [47]. In other words, the displays with the *less* striking pop-out effect give rise to the most *priming* of pop-out, and the displays with the most striking pop-out (as evidenced by faster overall RTs) give rise to smaller amounts of priming. A further observation regarding POP and DPE is that neither is observed when the task is simply to detect the presence/absence of a color pop-out in a display, which again implies that it is the assignment of colors to attention-roles (target/distractor) that underlies these effects. A final observation regarding POP is that, when one uses 3-item displays, the featural contrast between target and distractors on a given trial (i.e., how alike the target and distractor colors are) does *not* affect the magnitude of the priming effect, yet it does affect the overall RT, with faster overall RTs for larger color differences [75]. Thus, the magnitude of the color contrast appears to bear little to no relation to POP magnitude in three-items displays. This again suggests that POP is not a priming of “the phenomenon” of visual pop-out, but more likely reflects the attentional decision making “benefit” of repeating the same target/distractor color assignment across consecutive trials (a cognitive as opposed to perceptual effect). Thus, we propose that the “3-item pop-out” task is better understood as a “3-item attentional decision making” task.

In sum, the arguments above are meant to emphasize that the three-item display likely does not induce a purely bottom-up guidance of attention in the first place, which likely explains why Priming of Pop-out is so dependent on observers' goals and motivations [8], [35], [36]. We also believe that it is precisely because the task is an attentional decision making task that our model was able to successfully fit the data, and why it is that the logic of adaptation in bias applies in understanding the effect of selection history in POP and DPE so well.

### Limitations of the Current Modeling Approach

While our display had three objects, the Ratcliff diffusion model that we used models the competition between two choices, rather than three. That is, our model did not include the location decision that was made in the actual task. It is thus possible that the RDM can't fully capture the internal mechanisms of POP and the DPE. We made the choice to model color competition because we believe that the critical decision to make in a trial was whether the green color or red color was the target when an oddball was present [17], [20], [21]. Using a two-competitor model was likely acceptable for several reasons. First, Krajbich and Rangel [76] found that a model using three competitors had similar predictions of choice, reaction time and eye movements as a model using two competitors. They therefore suggested that the brain uses similar computation processes in the selection between two or among three choices. Moreover, they found that the resulting parameter values were comparable. Second, if the two-choice model was unable to describe our task, our study would not have yielded high quality model fits. Third, a behavioral signature of pop-out tasks is their independence of the number of items in the display, so it seemed contrary to the behavioral evidence to model the effect with three accumulators. That said, it will be important in the future to pursue modeling work to determine whether there are advantages to using a model with three accumulators, in particular whether this model can better determine the underlying mechanisms of POP and the DPE. One advantage may be modeling the role of location repetition in the oddball task [6], [7]. It may be possible to model color and location priming repetition effects concurrently to see if the color priming effects and the location priming effects are independent (see Maljkovic and Nakayama, 2000 [7]).

The RDM used here can only reflect *relative* bias; for instance, it does not dissociate whether the bias is towards a target or away from a distractor. However, it has been previously shown in many studies that POP can be due to dissociable effects on the target and distractors [17]–[19], [77], [78]. Models with additional free parameters, such as the leaky competing accumulator model [79], may be able to dissociate these effects in a modeling framework, and should be investigated in future work.

Another potential limitation of our modeling is our small sample size (*N* = 5). This was limited due to the large number of trials per subject. However, our main result that the bias parameter is critical for modeling both the DPE and POP is relatively consistent across subjects and is statistically significant (see *Modeling Results*). It would be valuable to increase the sample size in order to better understand the variation in other parameters across subjects. For instance, it is possible that there are additional parameter variations related to the DPE and POP that are found in just a fraction of the population.

### Significance

In our study, we used saccades as the response modality, allowing us to focus our analysis on the first act of selection on each trial. Note that all-but-two papers on Priming of Pop-out have used manual responses in the search task. The use of saccade latency as well as accuracy allowed us to model, for the first time, the attentional decision processes that underlie Priming of Pop-out and the Distractor Preview Effect, two robust inter-trial effects caused by changes in selection history. We importantly found that a diffusion model was able to accurately model these two effects. The modeling results allowed us to identify contributing factors to these two effects (thereby contributing to the literature on these two effects and selection history in general) and more generally, to further understanding of behavioral correlates of diffusion model parameters. The results are consistent with the idea that these two phenomena are not “visual priming” effects, but rather attentional priming effects, where the behavioral benefits/costs emerge from an attentional-decision making process that is tasked with determining, on every trial, the target/distractor assignments to the colors in the display.

## Supporting Information

Table S1 **Best-Fit Parameters of Expanded Model.**
(DOCX)

Table S2 **Best-Fit Parameters of Optimal Sub-Model.**
(DOCX)
