# Correction: Modeling the Effect of Selection History on Pop-Out Visual Search

**DOI:** 10.1371/journal.pone.0100049

**Published:** 2014-06-10

**Authors:** The PLOS ONE Staff

There is an error in the grant number associated with the National Science Council grant in the financial disclosure statement. The correct financial disclosure statement is:

“This work was funded with support from a grant # NSC 102-2410-H-006-019 from National Science Council (YCT, PI) and with a grant # BCS 07-46586 CAR from National Science Foundation (AL, PI). The funders had no role in study design, data collection and analysis, decision to publish, or preparation of the manuscript.”
