# Supplementary material for: Modeling the Effect of Selection History on Pop-Out Visual Search
Source: PLoS One. 2014 Mar 3;9(3):e89996. doi: 10.1371/journal.pone.0089996 (PMC3940711; doi:10.1371/journal.pone.0089996)
Supplement: Table S1 — Best-Fit Parameters of Expanded Model. (DOCX) [file pone.0089996.s001.docx]

|  | P1 | P2 | P3 | P4 | P5 | (Mean, SEM) |
| --- | --- | --- | --- | --- | --- | --- |
| **χ**^2^ | 7.844 | 11.997 | 22.019 | 3.209 | 14.240 | (11.862, 3.161) |
| **Δ***v*_DPE_ | -0.0516 | -0.0038 | 0.0351 | 0.0876 | 0.0103 | (0.0155, 0.0229) |
| **Δ***v*_POP_ | -0.0218 | 0.0397 | 0.1020 | -0.1338 | -0.0121 | (-0.0052, 0.0390) |
| **Δ***B*_DPE_ | 0.1648 | 0.1488 | 0.0828 | -0.0510 | 0.0709 | (0.0833, 0.0382) |
| **Δ***B*_POP_ | 0.1493 | 0.1008 | 0.0192 | 0.1384 | 0.0599 | (0.0935, 0.0243) |
| *a_DPE_* | 0.0597 | 0.0830 | 0.1353 | 0.0792 | 0.0913 | (0.0897, 0.0125) |
| *a_POP_* | 0.0640 | 0.0949 | 0.0841 | 0.0982 | 0.0959 | (0.0874, 0.0063) |
| *T_DPE_* | 0.2565 | 0.2515 | 0.2196 | 0.3305 | 0.2515 | (0.2619, 0.0184) |
| *T_POP_* | 0.2473 | 0.2421 | 0.2418 | 0.3073 | 0.2552 | (0.2587, 0.0124) |
| *v_0_* | 0.6930 | 0.6447 | 0.7488 | 0.6468 | 0.6630 | (0.6793, 0.0194) |
| *S_z_*_­_DPE_ | 0.0229 | 0.0000 | 0.0031 | 0.0479 | 0.0526 | (0.0253, 0.0109) |
| *S_z_*_­_POP_ | 0.0000 | 0.0710 | 0.0726 | 0.0000 | 0.0710 | (0.0429, 0.0175) |
| *S­_t_*_­_DPE_ | 0.0409 | 0.0708 | 0.0490 | 0.0845 | 0.0256 | (0.0542, 0.0105) |
| *S­_t_*_­_POP_ | 0.0491 | 0.0501 | 0.0452 | 0.0940 | 0.0151 | (0.0507, 0.0126) |
| *η*_DPE_ | 0.0788 | 0.2111 | 0.4795 | 0.1294 | 0.0000 | (0.1798, 0.0824) |
| *η*_POP_ | 0.2270 | 0.0000 | 0.5000 | 0.1958 | 0.0000 | (0.1846, 0.0921) |
