# Supplementary material for: Modeling the Effect of Selection History on Pop-Out Visual Search
Source: PLoS One. 2014 Mar 3;9(3):e89996. doi: 10.1371/journal.pone.0089996 (PMC3940711; doi:10.1371/journal.pone.0089996)
Supplement: Table S2 — Best-Fit Parameters of Optimal Sub-Model. (DOCX) [file pone.0089996.s002.docx]

|  | P1 | P2 | P3 | P4 | P5 | (Mean, SEM) |
| --- | --- | --- | --- | --- | --- | --- |
| **χ**^2^ | 9.744 | 14.958 | 25.050 | 9.613 | 19.548 | (15.783, 2.960) |
| **Δ***B*_DPE_ | 0.1478 | 0.1492 | 0.1119 | 0.0066 | 0.0684 | (0.0968, 0.0269) |
| **Δ***B*_POP_ | 0.1364 | 0.0981 | 0.0861 | 0.0592 | 0.0594 | (0.0878, 0.0143) |
| *a_DPE_* | 0.0566 | 0.0747 | 0.1114 | 0.1180 | 0.0927 | (0.0907, 0.0114) |
| *a_POP_* | 0.0643 | 0.0760 | 0.0763 | 0.1640 | 0.0933 | (0.0948, 0.0179) |
| *T_DPE_* | 0.2575 | 0.2496 | 0.2314 | 0.3374 | 0.2531 | (0.2658, 0.0184) |
| *T_POP_* | 0.2466 | 0.2420 | 0.2416 | 0.3097 | 0.2540 | (0.2588, 0.0129) |
| *v_0_* | 0.6906 | 0.5091 | 0.6728 | 1.2351 | 0.6532 | (0.7522, 0.1249) |
| *S_z_*_­_ | 0.0000 | 0.0000 | 0.0622 | 0.1059 | 0.0635 | (0.0463, 0.0205) |
| *S­_t_*_­_ | 0.0467 | 0.0726 | 0.0473 | 0.0871 | 0.0159 | (0.0539, 0.0122) |
| *η* | 0.2192 | 0.0007 | 0.4086 | 0.4999 | 0.0000 | (0.2257, 0.1025) |
